# Notes and News

**Published:** 1893-03-18

**Authors:** 


### Notes and News.

COLLECTED AND COMMUNICATED.

In one part of America endeavours are being made to pass a Bill requiring that all secret proprietary medicines containing certain poisonous substances shall be labelled with the name, quantity, and antidote of these poisons. It is not considered very probable that the Bill will get through.

It would seem to be the wise practice in America to increase the resources of existing hospitals by the addition of special departments, instead of establishing the less satisfactory small special institutions. At Chicago an orthopedic service has been added to St. Luke's Free Hospital.

Lord Justice A. L. Smith has just given judgment that the Royal College of Physicians is entitled to grant diplomas in surgery as well as in medicine. He argued that the word medicine embraced the whole art of healing, and moreover that the Royal College of Physicians being empowered to examine in surgery was entitled to grant diplomas also.

The subject of the Cholera Hospital for London seems likely to cause a good deal of trouble for some time to come. Mr. Ernest Hart has thrown the weight of his opinion on the side of those who oppose the floating site on the Thames. Granting that cholera is entirely a water-borne disease, he considers that a waterway is the last place that should be chosen.

The annual general meeting of the Royal Westminster Ophthalmic Hospital was held on the 6th inst. at the Mansion House, the Lord Mayor presiding. Sir Charles A. Turner, K.C.I.E., was elected Chairman of Committee, in place of Sir Rutherford Alcock, who has been compelled to resign on account of ill-health. The committee are seriously contemplating the rebuilding of the hospital on a new and much larger site. The present building is now sixty-two years old, and is totally inadequate to meet the constantly increasing demands that are made upon it. £50,000 will, it is estimated, be required, and the Lord Mayor earnestly appealed to those present and to the wealth and intelligence of the City of London to help the committee in every way possible in their efforts to raise the required sum.

Professor Horsley has been discoursing on hypnotism at the Royal Institution. He argues that hypnotism is simply mesmerism with a high light thrown upon it, and that the professed power of one will over another is attained by creating fatigue of the nerve centres and thus inducing catalepsy. Such derangement of the nervous system is a serious matter, and Professor Horsley is of the opinion that the exercise of hypnotism should be restricted by law.

The twelfth German Congress of International Medicine will be held in Wiesbaden during the third week in April. Amongst the subjects to be discussed are cholera and traumatic neuroses. A large number of papers on different subjects are to be read. We are not aware whether it is permissible for foreigners to read papers in their own language. German is not one of the official languages to be used at the Pan-American Congress, and on this account a distinguished German surgeon declined an invitation to participate.

The most extraordinary statement as respecting the financial arrangements of a hospital and its secretary, Mr. Kenyon Benham, has been made in evidence during the inquiry into the affairs of the London and General Bank. It appeared that Mr. Benham first opened an account with the bank for his hospital in 1887. At the end of 1889 he owed the bank upwards of £10,800, and in 1891 the debt had increased to £47,000. It appears that the bills offered by Benham for discount were never submitted to the committee in the ordinary course, and all these bills were dishonoured at maturity. A more discreditable disclosure has never been made in connection with any hospital. Was there any hospital in existence to which Mr. Kenyon Benham was attached? It would appear not, as the name of the hospital was not mentioned, and we have failed to obtain it. No management would countenance so huge an overdraft in so short a time, and it is hardly likely that the money was ever devoted to hospital purposes.

The work of the Samaritan societies connected with our hospitals is very highly valued by those who, through practical knowledge, are acquainted with the frequently dire needs of patients beyond and added to medical treatment. Some hospitals especially are situated in such poor and crowded neighbourhoods that it is with reluctance that the officials of the institutions dismiss patients, knowing as they do that their circumstances and surroundings must retard recovery, and not infrequently cause them to return to the wards after but a short absence. Samaritan societies are of gradual and latter growth, and very few strictly so-called exist. The hospitals have such urgent need of direct support that these auxiliary branches do not get the substantial assistance they certainly need and deserve. We are glad to learn that good friends arise from time to time, and that by the will of the late Mr. Henry Spicer the Samaritan societies of the London, the Middlesex, Guy's and St. Thomas's, the Metropolitan, and Royal Free Hospital become possessors of a munificent addition to their funds, which will probably amount to nearly £20,000 each.

